# Efficacy and safety of fire needle therapy for blood stasis syndrome of plaque psoriasis: protocol for a randomized, single-blind, multicenter clinical trial

**DOI:** 10.1186/s13063-020-04691-7

**Published:** 2020-08-25

**Authors:** Liu Liu, Yi Lu, Xiao-ning Yan, Su-qing Yang, Li-ping Gong, Ling-e Li, Yi-ding Zhao, Qing-feng Yin, Rui-ping Wang, Yue-peng An, Gang Huang, Jin-fang Zhang, Xiao-ying Sun, Xin Li, Bin Li

**Affiliations:** 1 Department of Dermatology, Yueyang Hospital of Integrated Traditional Chinese and Western Medicine, Shanghai University of Traditional Chinese Medicine, Shanghai, 200437 China; 2 Shanghai University of Traditional Chinese Medicine, Shanghai, 201203 China; 3 Department of Dermatology, Shaanxi Hospital of Traditional Chinese Medicine, Xi’an, 710003 Shannxi China; 4 Department of Dermatology, First Affiliated Hospital of Heilongjiang University of Traditional Chinese Medicine, Harbin, 150040 Heilongjiang China; 5 Department of Dermatology, The Affiliated Hospital of Jiangxi University of Traditional Chinese Medicine, Nanchang, 330006 Jiangxi China; 6 Department of Dermatology, Traditional Chinese Medicine Hospital of Shijiazhuang, Shijiahzuang, 050011 Hebei China; 7 Jiangsu Famous Medical Technology Co. Ltd, Nanjing University of Traditional Chinese Medicine, Floor 2, Building 19, Nanjing, 210029 China; 8 Office of Clinical Medical Research Center, Yueyang Hospital of Integrated Traditional Chinese and Western Medicine, Shanghai University of Traditional Chinese Medicine, Shanghai, 200437 China; 9 Institute of Dermatology, Shanghai Academy of Traditional Chinese Medicine, Shanghai, 201203 China

**Keywords:** Fire needle therapy, Plaque psoriasis, Blood stasis syndrome, Randomized controlled trial, Study protocol

## Abstract

**Background:**

Fire needle therapy is a characteristic treatment in traditional Chinese medicine (TCM). An increasing number of studies have indicated that fire needle treatment for psoriasis provides satisfactory results with few side effects and a low recurrence rate. We herein describe the protocol for a multicenter, randomized, single-blind, placebo-controlled trial that will provide high-quality evidence on the efficacy and safety of fire needle therapy for plaque psoriasis.

**Methods:**

Ninety-two patients with blood stasis syndrome (BSS) of plaque psoriasis will be enrolled and randomly assigned to receive fire needle therapy (intervention group) or fire needle control therapy (control group) once a week for 4 weeks. The Psoriasis Area and Severity Index (PASI) score will serve as the major efficacy index, while the body surface area (BSA), Physician Global Assessment (PGA) score, Dermatology Life Quality Index (DLQI) score, patient-reported quality of life (PRQoL), visual analog scale (VAS) score for itching, TCM symptom score, and relapse rate will be assessed as secondary outcomes. The PASI score, BSA, PGA score, and VAS score for itching will be evaluated at baseline and during the 4-week treatment and follow-up periods. DLQI score, PRQoL, and TCM symptom score will be assessed at baseline and during the treatment period. Recurrence will be evaluated during the follow-up period. Safety assessments include vital sign monitoring, routine blood tests, blood biochemistry, routine urine tests, pregnancy tests, physical examinations, and adverse-event recording. SAS software will be used for data analysis. The data network platform will be designed by the data management center of Nanjing Ningqi Medical Technology Co., Ltd.

**Discussion:**

It is believed that fire needle therapy can activate the meridians, promote blood circulation, and regulate skin immunity. BSS of plaque psoriasis is related to not only immune dysfunction but also poor or stagnant blood flow. We anticipate that the results of the trial described in this protocol will provide strong evidence for the safety and efficacy of fire needle therapy for BSS of plaque psoriasis.

**Trial registration:**

Clinicaltrials.govNCT03953885. Registered on May 15, 2019. Name: Fire Needle Therapy on Plaque Psoriasis with Blood Stasis Syndrome

## Background

Psoriasis is a chronic inflammatory skin disease characterized by red papules or plaques covered with silvery-white scales [1]. The pathogenesis is unclear but believed to involve interactions among genetic factors, the immune system, and the external environment [1, 2]. The global prevalence of this condition varies from 0.51 to 11.43% in adults and 0 to 1.37% in children [3]. In China, the prevalence is approximately 0.47% [4]. Current treatments primarily involve the use of biological agents such as T cell inhibitors, tumor necrosis factor-alpha (TNFα) inhibitors, interleukin (IL)-12/23 blockade, and other targeted therapies [1]. However, these targeted therapies are expensive and not effective in all cases. Consequently, the majority of patients resort to traditional Chinese medicine (TCM) therapy.

TCM is known for its unique theoretical system. TCM doctors use individualized treatments after syndrome differentiation in each case; these may include Chinese herbal medicine, acupuncture, and moving cupping therapy [5], among others. Fire needle therapy, a characteristic TCM therapy, is a form of acupuncture treatment. It involves the penetration of needles heated over alcohol lamps at the designated acupoints on the human body; based on TCM theory, this warms yang and expels cold, disperses stasis and relieves pain, and warms and activates the meridians [6]. In the clinical setting, fire needle therapy has proven effective for some diseases, such as moderate-severe acne [7] and nodular prurigo [8]. According to TCM theory, blood stasis syndrome (BSS), one of the basic syndromes of psoriasis, is caused by poor or stagnant blood flow [9]. A previous study showed that fire needle therapy is effective for plaque psoriasis in the stationary phase with few side effects and a low recurrence rate [10]. Similarly, a growing body of evidence has shown satisfactory clinical efficacy of fire needle therapy for plaque psoriasis [11]. However, to our knowledge, there is a dearth of multicenter, blinded studies using only fire needles and emollients for the treatment of psoriasis.

We herein present the protocol for a multicenter, randomized, single-blind, placebo-controlled trial for objective and standardized evaluations of the clinical efficacy and safety of fire needle therapy for plaque psoriasis. The aim of the trial is to obtain objective evidence in accordance with international standards and aid in the development of clinical norms that will facilitate the treatment’s popularization and clinical application.

## Methods/design

### Study setting

**Fig. 1:**
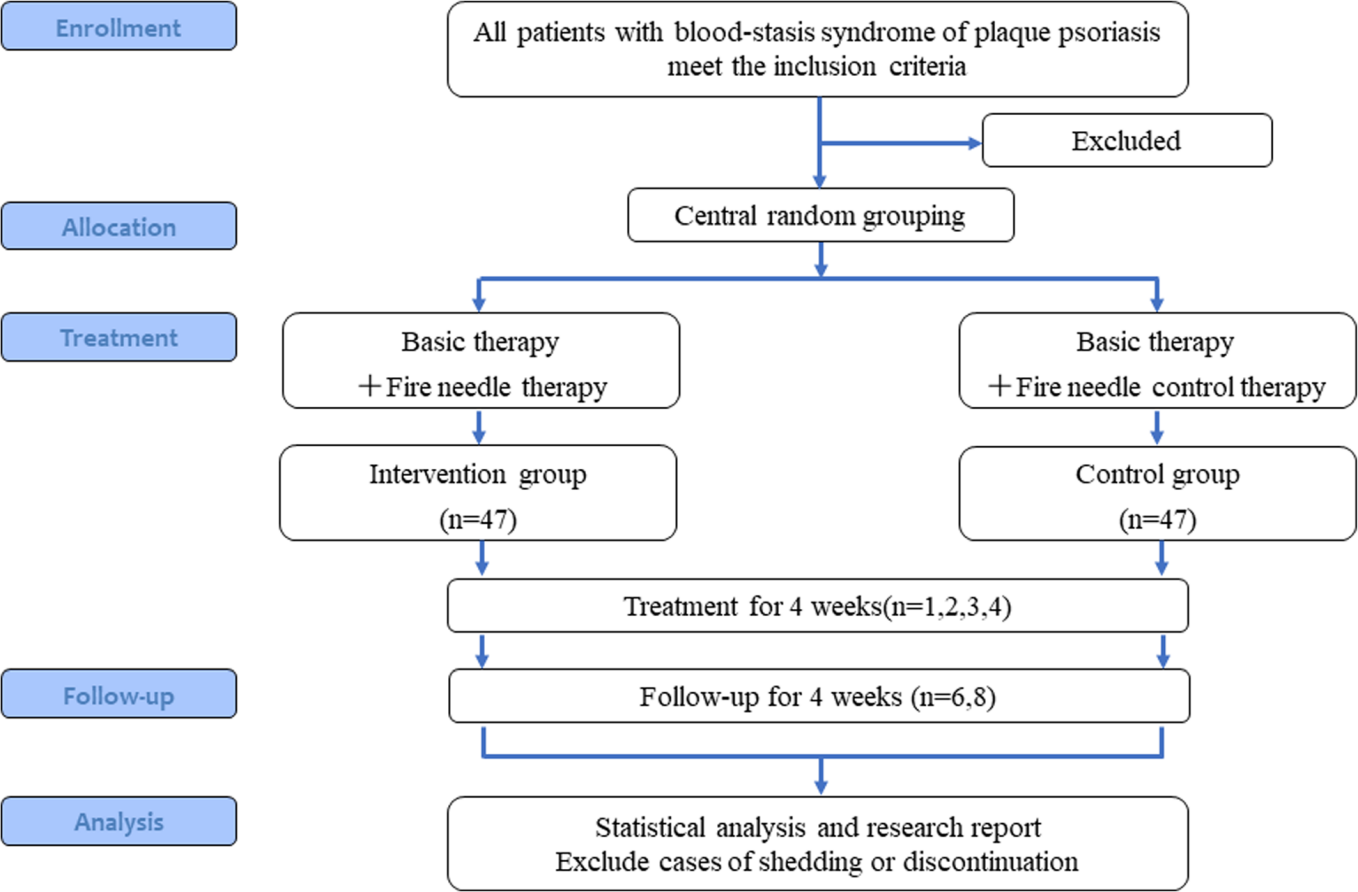
Study flowchart. This is the protocol for a randomized controlled trial on the efficacy and safety of fire needle therapy for blood stasis syndrome of plaque psoriasis

This is a multicenter, single-blind, randomized controlled trial to investigate the efficacy and safety of fire needle therapy for plaque psoriasis. The study will be performed in five centers in China: Yueyang Hospital of Integrated Traditional Chinese and Western Medicine, Shaanxi Hospital of Traditional Chinese Medicine, First Affiliated Hospital of Heilongjiang University of Traditional Chinese Medicine, Affiliated Hospital of Jiangxi University of Traditional Chinese Medicine, and Traditional Chinese Medicine Hospital of Shijiazhuang. Participants will be assigned to treatment and control groups using a centralized randomization system (implemented by a data network platform designed by Nanjing Ningqi Medical Technology Co., Ltd.). The detailed process is shown in a flowchart in Fig. 1.

This protocol has been developed according to the Standard Protocol Items: Recommendations for Interventional Trials (SPIRIT) 2013 guidelines [12]. This checklist is described in detail in Additional file 1. The protocol has been approved by the institutional review board (IRB) of Yueyang Hospital of Integrated Traditional Chinese and Western Medicine, Shanghai University of TCM (ethical approval number: 2019–033), and the study is registered at *clinicaltrials.gov* (ID: NCT03953885; registration date: May 15, 2019).

### Diagnostic criteria

In Western medicine, the diagnostic criteria for plaque psoriasis have been formulated in accordance with *Guideline for the diagnosis and treatment of psoriasis in China (2018 simplified edition)* [13], whereas those for BSS of plaque psoriasis have been drafted according to the *Evidence-based clinical practice guide for psoriasis vulgaris* by the Dermatology Branch of China Association of Chinese Medicine [14].

Accordingly, participants will be diagnosed with plaque psoriasis based on the following criteria [13]: First, the skin lesions appear as dark red plaques or invasive erythema with clear boundaries showing white or silvery-white scales. Second, features such as the “wax drop phenomenon,” “film phenomenon,” “point bleeding phenomenon” (Auspitz sign), and “bundle hair” can be found during physical examination. Third, the scalp, back, and extremities are the most susceptible areas for skin lesions. Fourth, itching may or may not be present. Fifth, the Koebner phenomenon can be observed in the advanced stage. Finally, the skin lesions are prone to relapse, with the majority alleviated in summer and aggravated in winter.

The main sign of BSS of plaque psoriasis is the prolonged presence of dark red, hypertrophic, infiltrating skin lesions. Minor signs include scaly dry skin, a soot-black facial complexion, cyanosis of the lips and nails, dark menstrual blood or blood clots in female patients, a dark purple tongue with ecchymosis and/or petechiae, an unsmooth pulse, and a thready/moderate pulse. The presence of BSS will be confirmed when patients show the main sign along with more than one minor sign [14, 15].

### Inclusion and exclusion criteria

Participants will be recruited from five hospitals in China: Shaanxi Hospital of Traditional Chinese Medicine, Yueyang Hospital of Integrated Traditional Chinese and Western Medicine, First Affiliated Hospital of Heilongjiang University of Traditional Chinese Medicine, Affiliated Hospital of Jiangxi University of Traditional Chinese Medicine, and Traditional Chinese Medicine Hospital of Shijiazhuang. Each research center has one or two clinical research observers (CRO) who will explain this trial to participants before they voluntarily sign an informed consent form at the beginning of the study. On the consent form, participants will be asked if they agree to the use of their data, should they choose to withdraw from the trial. Participants will also be asked to grant permission for the research team to share relevant data with the universities taking part in the research or regulatory authorities, where relevant. This trial does not involve the collection of biological specimens for storage.

The general inclusion criteria for the study are as follows: (1) compliance with the abovementioned diagnostic criteria for plaque psoriasis and BSS of plaque psoriasis; (2) skin lesions involving ≤ 10% of the body surface area (BSA), with primary involvement of the trunk and/or limbs, palms/soles, and/or face/scalp (vulva not included); (3) aged 18 to 65 years (inclusive of 18 and 65 years); and (4) provision of written informed consent for participation in the study.

Exclusion criteria will be applied as follows: (1) presence of other progressive skin diseases that may affect assessment of the condition; (2) treatment with the research drugs, biological agents, and immunosuppressive agents within 1 month before study initiation; (3) treatment with topical glucocorticoids and/or phototherapy within 2 weeks before study initiation; (4) presence of severe, uncontrolled, local or systemic, acute or chronic infection; (5) presence of severe systemic diseases, elevation of alanine aminotransferase or aspartate aminotransferase levels by > 1.5 times the upper limit of normal, elevation of creatinine by 1.5 times the upper limit of normal, decrease in any main blood test parameters (white blood cell count, red blood cell count, hemoglobin amount, platelet count) to below the lower limit of normal, or abnormalities in other laboratory test parameters; (6) history of malignant neoplasms, primary or secondary immune deficiency, and/or a hypersensitivity reaction; (7) major surgery within 8 weeks before study initiation or scheduled during the study period; (8) pregnancy or lactation; (9) history of alcohol abuse or drug abuse, family history of cancer, personal or family history of a serious mental illness; (10) any other factors considered by the researchers as contraindications for study participation.

Criteria for trial suspension, patient rejection, and patient withdrawal are also set as follows: (1) development of other serious or life-threatening organic lesions during the trial, (2) withdrawal at the individual’s discretion, (3) poor patient adherence to treatment, (4) participation in another trial administering treatment that may interfere with the results of the present study, (5) refusal to take contraceptive measures during the trial period (including both treatment and follow-up periods), (6) loss to follow-up for various reasons, and (7) incorrect application of the inclusion and exclusion criteria.

### Randomization and masking

This trial will follow a randomized, single-blind design. The data management company will use a centralized randomization system to generate random codes, which will be sent to the project lead unit. The project lead unit will prepare a random code table and distribute it to the participating units. The random code will then be distributed to the participants by the CRO of each participating unit. Only the CRO responsible for patient recruitment will be aware of the group allocation until the participants have completed the trial. All other research staff, including the individual responsible for measurements, will remain blinded to the group allocation. If participants know which group they belong to, they will be instructed to refrain from sharing the information with the individual responsible for measurements. The participants will be informed of their results only after completion of recruitment, interventions, and evaluations for all participants.

### Interventions

Patients will be randomly assigned to receive fire needle therapy (intervention group) or fire needle control therapy (control group) once a week for 4 weeks. In addition, all patients will receive basic treatment, including prescription of a moisturizing lotion, instructions to avoid inducing and aggravating factors, instructions to bathe appropriately, and lifestyle recommendations.

#### Fire needle group

The participants in this group will wear eye masks and fully expose the lesions. After selecting the acupoints, the operator will routinely disinfect his/her hands and the acupuncture points on the participants. Then, the operator will select an acupuncture needle measuring 0.4 × 40 mm, ignite the alcohol lamp, and continuously move the needle from its root to its tip in the outer flame until the needle turns red; this is for disinfection of the needle. After disinfection, the operator will heat the needle tip and body in the outer flame until it turns completely red (the length of the red portion is determined by the acupuncture depth) and rapidly penetrate the skin lesion in a vertical direction; the depth of the acupuncture is based on the thickness of the skin lesion. Puncture will be performed from the lesion periphery to the lesion center at intervals of 0.3 to 0.5 cm.

#### Fire needle control group

In this group, the operators will directly penetrate the skin lesion without heating the needle. All other procedures will remain the same as those described for the intervention group.

After treatment, all patients will receive an explanation that redness, warmth, mild pain, and/or itching at the acupuncture sites are normal side effects that will resolve on their own. Moreover, they will be instructed to maintain cleanliness at the acupuncture sites, avoid scratching with their hands, refrain from using oils or creams, and refrain from exposing the punctured sites to water on the day of treatment.

### Outcomes

#### Primary outcomes

The Psoriasis Area and Severity Index (PASI) is the gold standard assessment tool used to quantify the severity of psoriasis based on erythema, infiltration, scaling, and the extent of lesions [16]. As such, a decrease in the PASI score has been designated as the primary outcome of this study. This score will be evaluated at screening, at baseline, during the 4-week treatment period, and during the 4-week follow-up period. The researchers will use the PASI to assess the severity and area of lesions and simultaneously obtain photographs of typical skin lesions.

#### Secondary outcomes

Secondary outcomes will include the BSA and Physician Global Assessment (PGA) score to describe the severity of psoriasis. The BSA will also be used to estimate the body area of psoriasis lesions, and PGA scores will be used to compare the baseline conditions of patients with psoriasis [17]. As itching is a common symptom of psoriasis, a visual analog scale (VAS) will be used to evaluate the degree of itching. Apart from these, we will also use two tools to assess the quality of life of patients with psoriasis: The Dermatology Life Quality Index (DLQI) [18] and the patient-reported quality of life (PRQoL). TCM symptoms will be assessed using TCM symptom scores, which are formulated according to TCM diagnostic criteria. When scoring, patients only need to answer “yes” or “no”; “yes” counts as 1 point, “no” counts as 0 points, and the points are combined to calculate the total score (Table 1). The recurrence rate will also be calculated to evaluate the efficacy of fire needle therapy.

**Table 1:** TCM symptom scores for blood stasis syndrome of plaque psoriasis

Symptoms	Scores	
The prolonged presence of dark red skin lesions	0, No	1, Yes
The prolonged presence of hypertrophic, infiltrating skin lesions	0, No	1, Yes
Scaly dry skin, a soot-black facial complexion, cyanosis of the lips and nails	0, No	1, Yes
Dark menstrual blood or blood clots in female patients	0, No	1, Yes
A dark purple tongue with ecchymosis and/or petechiae	0, No	1, Yes
An unsmooth pulse and a thready/moderate pulse	0, No	1, Yes
Total scores		

The BSA, PGA, and VAS scores will be evaluated along with the PASI score at baseline and during the treatment and follow-up periods. The PRQoL, DLQI, and TCM symptom scores will be assessed at baseline and during the 4-week treatment period, while recurrence will be assessed during the follow-up period. For this study, recurrence is defined as a follow-up PASI score exceeding the score at the time of enrollment, the appearance of new pustules during the follow-up period, or the development of erythroderma during the follow-up period. Once recurrence is confirmed, the participant will be given the option of terminating the follow-up and initiating other treatments.

#### Safety

Safety assessments will include vital sign monitoring, routine blood tests, blood biochemistry, routine urine tests, pregnancy tests, physical examinations, and the recording of adverse events (including serious adverse events). Participants will be monitored for their vital signs, adverse events, and serious adverse events throughout the research period. Routine blood and urine tests and blood biochemistry will be conducted at the time of screening and during the 4th week of treatment. Pregnancy tests will be conducted only at the time of screening. Physical examinations will be conducted at the time of screening, during the 4th week of treatment, and throughout the follow-up period (Table 2).

**Table 2:** Timeline for a randomized trial evaluating fire needle therapy for blood stasis syndrome of plaque psoriasis

	Research cycle	Screening period	Baseline period	Treatment period				Early termination	Follow-up period	
	Time point	Week 1	Week 0	Week 1	Week 2	Week 3	Week 4		Week 6	Week 8
**Enrollment**	**Inclusion/exclusion criteria**	**●**	**●**							
	**Informed consent**	**●**								
	**Demographic characteristics**	**●**								
	**Medical history**	**●**								
	**Registration**	**●**								
	**Randomization**		**●**							
	**Photographs of skin lesions**		**●**	**●**	**●**	**●**	**●**	**●**	**●**	**●**
**Interventions**	**Basic therapy + fire needle therapy**		**★-------------------------------------------------------------------★**							
	**Basic therapy + fire needle control therapy**		**☆-------------------------------------------------------------------☆**							
**Assessment**	**PASI**	**●**	**●**	**●**	**●**	**●**	**●**	**●**	**●**	**●**
	**BSA**	**●**	**●**	**●**	**●**	**●**	**●**	**●**	**●**	**●**
	**PGA**	**●**	**●**	**●**	**●**	**●**	**●**	**●**	**●**	**●**
	**DLQI**		**●**	**●**	**●**	**●**	**●**	**●**		
	**PRQoL**		**●**	**●**	**●**	**●**	**●**	**●**		
	**VAS**		**●**	**●**	**●**	**●**	**●**	**●**	**●**	**●**
	**TCM symptom**		**●**	**●**	**●**	**●**	**●**	**●**		
	**Recurrence rate**								**●**	**●**
**Safety**	**Vital signs**	**●**	**●**	**●**	**●**	**●**	**●**	**●**	**●**	**●**
	**Blood routine**	**●**					**●**	**●**		
	**Blood biochemistry**	**●**					**●**	**●**		
	**Urine routine**	**●**					**●**	**●**		
	**Concomitant medication**	**●**	**●**	**●**	**●**	**●**	**●**	**●**		
	**Pregnancy test**	**●**								
	**Physical examination**	**●**					**●**	**●**	**●**	**●**
	**Adverse events**		**●**	**●**	**●**	**●**	**●**	**●**	**●**	**●**
	**Serious adverse events**	**●**	**●**	**●**	**●**	**●**	**●**	**●**	**●**	**●**

*PASI* Psoriasis Area and Severity Index, *BSA* body surface area, *PGA* Physician Global Assessment, *DLQI* Dermatology Life Quality Index, *PRQoL* patient-reported quality of life, *VAS* visual analog scale, *TCM* traditional Chinese medicine

★: Fire needle therapy group

☆: Fire needle control therapy group

### Sample size

In this study, the estimated post-treatment PASI score for all patients will be set at 50% lower than the pre-treatment score, both in the intervention group and the control group. The sample size of the current trial is calculated based on the following formula, and take the cure rate as the primary treatment effect index.

$$ n=\frac{p_1\times \left(1-{p}_1\right)+{p}_0\times \left(1-{p}_0\right)}{{\left({p}_1-{p}_0\right)}^2}{\left({z}_{\alpha /2}+{z}_{\beta}\right)}^2 $$

According to a previous study titled “Therapeutic Observation of Fire-needle Acupuncture for Stationary Plaque Psoriasis” [19], the cure rate to achieve this score is 76.8% for the intervention group and 35.3% for the control group. In this study, we set the inspection level (*α*) as 0.05, and the test power as 0.9, then *β* = 1–0.9 = 0.1. For two-sided tests, at least 25 participants would be required for each group. Considering the correlation of primary outcomes at different times and multicenter effect, we increase the sample size to 38 participants in each group, Considering a dropout rate of 20%, a total of 94 patients (47 per group) will be required for this study (Table 3).

**Table 3:** Centers involved in a randomized trial evaluating fire needle therapy for blood stasis syndrome of plaque psoriasis

Research center	Sample size
Shaanxi Hospital of Traditional Chinese Medicine	20
Yueyang Hospital of Integrated Traditional Chinese and Western Medicine	20
First Affiliated Hospital of Heilongjiang University of Traditional Chinese Medicine	18
Affiliated Hospital of Jiangxi University of Traditional Chinese Medicine	18
Traditional Chinese Medicine Hospital of Shijiazhuang	18

### Quality assurance system

The steering committee, composed of principal investigators (PI) from each sub-center, is responsible for monitoring the progress of the trial, training researchers before the study begins, reviewing adverse events, and developing statistical analysis programs with a statistician. Each sub-center has a clinical research assistant (CRA), and the five of them form the data monitoring committee. CRAs are responsible for checking all documents, such as case report forms (CRFs), related to this trial and coordinating all aspects of local organization. Each committee meets annually to review the trial process, and the interim analysis will take place in the second year of the study; the five research units will share the analysis results. Monitoring will be conducted independently from the PI and sponsors.

If the protocol is to be revised, it must be approved by the IRB of each research center, and the project lead unit may terminate all studies early due to medical reasons. In addition, if the research cannot be carried out in accordance with the research protocol agreement or if any other reason is found, the project lead unit has the right to terminate the research at any time. If the research is terminated or suspended early, the project lead unit should immediately notify the CRO, medical institution, management department, and IRB of the branch center of the decision and its reasons. All research materials must be returned to the project lead unit.

### Safety monitoring

#### Adverse events

Adverse events (AEs) are defined as any symptoms, syndromes, or diseases that affect the patient’s health during the observation period of this clinical study and also involve clinically relevant events found in the laboratory or other diagnostic processes. Examples include the need for unplanned treatment measures which result in patients withdrawing from the study, or when clinically meaningful laboratory test items are abnormal. Serious adverse events (SAEs) consist of (1) death, (2) life-threatening events, (3) hospitalization, (4) permanent or severe disability after fire needle therapy, (5) events leading to congenital malformations after fire needle treatment, and (6) the need for medical treatment to prevent permanent injury or damage. Fire needle therapy is considered safe, and no obvious AEs or SAEs have been reported in clinical application.

#### Management of AEs and SAEs

Researchers should always carefully observe the patient’s skin lesions and systemic performance during fire needle treatment. If any AEs occur during treatment, the researcher should complete the “adverse event form” within the CRF and conduct a follow-up investigation; they should record the treatment process in detail until the laboratory test is normal and the symptoms and signs disappear. In addition, once AEs occur, the researcher should make a diagnosis, initiate treatment according to the condition, and decide whether to stop the clinical observation. In the event of SAEs, the unit undertaking the clinical research must immediately take the necessary measures to protect the safety of the participants. The researcher will then complete the “Serious Adverse Event Report Form” within the CRF. He or she will report to the subject responsible unit as well as the ethics committee of the unit within 24 h and sign and date the report. The research responsible unit will report the SAEs to the participating units in a timely manner and ensure that the reporting procedures meet all laws and regulations. Once the steering committee determines whether the AEs or SAEs are related to this study, the research team will provide treatment costs and financial compensation for trial-related damages.

### Data confidentiality and management

With the exception of the CRO responsible for the participants, participants’ information will be kept confidential from research staff until publication of the study results. All of their documents related to this trial, such as CRFs, will be represented by the participant’s unique random code. Random codes and CRFs will be sealed and stored in a separate filing cabinet. To facilitate data analysis, CRO will also use a unified data collection system for recording (established by Nanjing Ningqi Medical Technology Co., Ltd. Data Management Center, Nanjing, China). The results of this trial will be published in an international peer-reviewed journal.

### Statistical analysis

A statistician will formulate a plan for statistical analysis in consultation with the PI. All analyses will be conducted using SAS statistical software, and the data network platform will be designed by the data management center of Nanjing Ningqi Medical Technology Co., Ltd., which will be entrusted with the third-party statistical analyses. The measurers will be blinded to the results. The overall principles will be as follows: An intention-to-treat (ITT) and per-protocol analysis will be used for statistical analysis. Quantitative variables (such as PASI, BSA, PGA, DLQI, PRQoL, VAS, and TCM syndrome scores) will be expressed as mean and standard deviation values or as median values with lower (Q1) and upper (Q3) quartiles. If the quantitative variables conform to normal distribution, a *t* test will be used (The homogeneity test will be performed for the two groups, with 0.05 as the significance level. The Satterthwaite method will be used to correct the *t* test when the variance is not uniform). If quantitative variables conform to skewed distribution, a nonparametric test will be used. Qualitative variables (such as cure rate, recurrence rate) will be expressed as numbers and percentages or composition ratios. Statistical inference will be performed using chi-square tests, Fisher’s exact tests, and Wilcoxon rank sum tests, among others. Potential confounding factors affecting the main outcome indicators will be adjusted by logistic regression. In this study, a *p* value of less than 0.05 is considered as statistically significant.

To account for missing data, we considered participants who may not complete the follow-up when calculating the sample size. The reasons, main characteristics, and data of a patient’s failure to follow-up will be recorded. Other types of randomly missing data will be considered unsuccessful according to the principle of ITT analysis.

## Discussion

The management of psoriasis has always been a focus of research in the field of dermatology. Dermatologists have continuously been exploring better ways to treat psoriasis, such as fish oil [20] and moving cupping therapy [5]. Professor Wan-zhang Qin, a famous Chinese medicine practitioner, put forward the “new blood syndrome theory” based on the assumption that psoriasis is centered on “blood.” According to this theory, blood-heat occurs first, followed by blood-deficiency, blood-dryness, and blood-coldness. Blood-poison represents malignant development of disease, and blood-stasis is present throughout the course of the disease [21]. In light of Qin’s theory, blood-stasis can be caused by factors such as blood-heat and blood-dryness. This shows that the long-term existence of blood-stasis is the main pathological factor for the chronicity of psoriasis. The essence of BSS is reportedly a hypercoagulable state manifested by blood circulation and microcirculatory disorders [22].

Fire needle therapy is a special acupuncture treatment involving the use of needles and moxibustion [6]. When the heated needle penetrates the skin, it can improve the local microcirculation and regulate cell metabolism. The stimulation of acupoints in accordance with the meridian conduction pathways can resolve both internal and external discomfort and regulate the balance between yin and yang in the organs [23]. At the same time, the skin receives warm stimuli that also promote blood circulation [6]. Further, skin immunity plays a big role in the development of psoriasis. A study showed that patients with blood-heat syndrome of psoriasis vulgaris had substantially elevated levels of interferon-gamma, IL-17, IL-23, and TNF-α and significantly decreased levels of IL-4 and IL-10 [24]. According to our recent study and other studies, fire needle therapy can inhibit inflammatory reactions by inhibiting the abnormal proliferation of T cells (such as CD8+T cells) and reducing the number of IL-2 and IFNγ; in addition, it can increase the number of CD4+T cells, IL-4, IL-10 and the ratio of CD4+T cells and CD8+T cells; and also, it can promote the balance of Th1 and Th2 cells by reducing the number of Th1 cells and increasing the number of Th2 cells to improve the condition of psoriasis [25–27].

In China, fire needle therapy is listed as a special TCM therapy in the *Guideline for the diagnosis and treatment of psoriasis in China (2018 simplified edition)* [13]. However, to the best of our knowledge, there is no multicenter clinical study on the treatment of psoriasis with fire needle therapy. Moreover, research involving only basic treatments and fire needle therapy is scarce. Accordingly, the results of the proposed randomized controlled trial on the efficacy and safety of fire needle therapy for BSS of plaque psoriasis are expected to provide a high level of evidence.

### Trial status

This is version 3.0, and the protocol was registered at *clinicaltrials.gov* on May 15, 2019. Participant recruitment was initiated in June 2019 and is scheduled to be completed by the end of December 2020.

## Supplementary information

**Additional file 1.** SPIRIT checklist (2013).

## Data Availability

The data and datasets analyzed during the current study are available from the corresponding author on reasonable request. Informed consent forms are also available from the corresponding author on request.

## Abbreviations

BSA: Body surface area
CRA: Clinical research assistant
CRO: Clinical research observer
DLQI: Dermatology Life Quality Index
PI: Principal investigator
IL: Interleukin
IRB: Institutional review board
ITT: Intention-to-treat
PASI: Psoriasis Area and Severity Index
PGA: Physician Global Assessment
PRQoL: Patient-reported quality of life
TCM: Traditional Chinese medicine
TNF: Tumor necrosis factor
SPIRIT: Standard Protocol Items: Recommendations for Interventional Trials
VAS: Visual analog scale
